# Knowledge, Attitude, and Practices of Paediatricians in the West Bank, Palestine, Regarding COVID-19 Vaccination Among Children Younger than 12 Years: A Cross-Sectional Study, October to November 2023

**DOI:** 10.3390/vaccines13121236

**Published:** 2025-12-11

**Authors:** Yousef Mosleh, Kostas Danis, Pawel Stefanoff, Diaa Hjaija

**Affiliations:** 1 Preventive Medicine Department, Palestinian Ministry of Health (PMOH), Ramallah P6009262, Palestine; pmd@moh.ps; 2 Mediterranean and Black Sea Programme in Intervention Epidemiology Training (MediPIET), European Centre for Disease Prevention and Control (ECDC), 171 83 Stockholm, Sweden; kostas.danis@ecdc.europa.eu (K.D.); pawel.stefanoff@ecdc.europa.eu (P.S.)

**Keywords:** COVID-19, vaccine hesitancy, paediatricians, knowledge, attitudes, practices, Palestine

## Abstract

**Background/Objectives**: Paediatricians’ recommendations influence parental decisions to vaccinate their children. On 19 January 2022, the World Health Organization authorized the Pfizer-BioNTech COVID-19 vaccine (BNT162b2) under Emergency Use Listing for children under 12 years as a measure to mitigate disease spread and direct protection for children with underlying conditions. We assessed knowledge, attitudes, and practices (KAP) of Palestinian paediatricians regarding COVID-19 vaccination for children under 12 years and identified factors affecting support for vaccination. **Methods**: From 1 October to 8 November 2023, we surveyed paediatricians across the West Bank using structured telephone interviews. We collected data on sociodemographic characteristics and KAP regarding COVID-19 vaccination and calculated KAP scores from eight, nine, and nine items, respectively, with total scores categorized as poor/moderate/good. We performed bivariable and multivariable analyses to identify factors associated with paediatricians supporting COVID-19 vaccination for children under 12 years. **Results**: Of the 367 eligible paediatricians, 323 (88%) responded; the median age was 51 years (range: 28–70); 27% supported COVID-19 vaccination for children. Mean scores for knowledge (range 0–8), attitude (0–9), and practice (0–9) were 3.0 ± 2.1, 3.9 ± 2.4, and 4.0 ± 1.7, respectively. The mean overall KAP score (0–26) was 11 ± 4.8. Safety and efficacy concerns and lack of long-term data were the main reasons for hesitancy. Higher knowledge scores (PR = 1.8, 95% CI: 1.3–2.5, *p* = 0.001) and positive attitudes (PR = 1.6, 95% CI: 1.1–2.3, *p* = 0.01) were significantly associated with paediatricians’ support for vaccination. After adjustment for other factors, participants with regular continuing medical education attendance (aPR = 1.4, 95% CI: 1.0–2.6, *p* = 0.045), trusting WHO recommendations (aPR = 3.1, 95% CI: 1.4–7.8, *p* = 0.047), having a positive attitude score (aPR = 1.3, 95% CI: 0.4–4.4, *p* = 0.041), and a good total KAP score (aPR = 1.1, 95% CI: 1.0–1.2, *p* = 0.044) supported COVID-19 vaccination for children. **Conclusions**: Support for COVID-19 vaccination among Palestinian paediatricians was low, associated with their knowledge, attitudes, and trust in health authorities. The revised WHO recommendations from 10 November 2023, decreasing the priority of vaccinating healthy children, could influence the opinion of paediatricians. However, the low support for COVID-19 vaccinations could affect the performance of other vaccination programmes and should be carefully addressed through targeted education.

## 1. Introduction

The COVID-19 pandemic placed a major burden on health systems worldwide, with millions of cases and deaths having been reported globally among adults and children [1,2]. Although children generally experience less severe acute illness and fewer deaths compared with adults, they may face serious health outcomes such as hospitalisation, multisystem inflammatory syndrome (MIS-C), and persistent symptoms known as post-COVID syndrome (Long COVID) [3,4,5]. Moreover, children and adolescents may contribute to community transmission of the virus [6].

The development and deployment of COVID-19 vaccines represented a cornerstone of the global strategy to control the pandemic. Vaccination aimed to reduce severe disease, transmission, and the emergence of new variants. mRNA COVID-19 vaccines demonstrated high efficacy (>90%) against severe disease and hospitalisation in children aged 5–11 years, and their deployment was considered highly cost-effective even under scenarios of lower viral circulation by preventing severe illness, MIS-C, and long-term complications [7]. In December 2020, the WHO granted Emergency Use Listing (EUL) for the Pfizer-BioNTech COVID-19 vaccine (BNT162b2) for individuals aged 16 years and older. This decision was based on data demonstrating the vaccine’s safety and efficacy in this age group. Subsequently, in 2021, the EUL was extended to include children aged 6 months to 15 years [8,9]. However, in the Strategic Advisory Group of Experts SAGE Roadmap for Prioritizing Uses of COVID-19 Vaccines published on 10 November 2023, the WHO revised its position, classifying healthy children and adolescents aged 6 months to 17 years as a low-priority-use group. The rationale was that more than three years into the pandemic, infection-induced and hybrid immunity had become widespread, and COVID-19-related deaths among healthy children had become rare, occurring mainly in those with comorbidities [10].

Although large clinical studies have demonstrated the efficacy and safety of COVID-19 vaccines in children, parents’ hesitancy and scepticism have remained a major barrier to achieving widespread coverage and controlling the spread of the virus globally [11,12]. Numerous studies exploring public acceptance demonstrated a high degree of hesitancy, mostly due to the belief that COVID-19 did not cause severe disease in children or concerns about vaccine safety, including the preference among a notable proportion of parents for their child to gain immunity through infection rather than vaccination [13,14,15,16]. In this context, studies reported that vaccine hesitancy among healthcare workers (HCWs) themselves negatively impacted their probability of recommending the vaccine to patients [17].

In Palestine, in August 2021, as the COVID-19 vaccine became more available, the Ministry of Health (MoH) extended the free-of-charge vaccination campaign to adolescents aged from 12 to 17 years. The purpose was to vaccinate this population prior to the start of the academic year, ensuring a safer return to schools during the ongoing pandemic. However, vaccine uptake had been critically low, with MoH data indicating that fewer than 20% of 12–17-year-olds in the West Bank received a primary series by late 2022, despite the availability of the vaccine (Palestinian Ministry of Health, 2024). The Ministry of Health initially followed the earlier WHO guidance and allowed COVID-19 vaccination for children aged 5 years and above. During the study planning and implementation, the WHO authorized the COVID-19 vaccination of healthy children under 12 years of age. Therefore, the Palestinian Ministry of Health had planned to expand the COVID-19 vaccination programme to include children under 12 years of age as part of the second stage of the school-aged vaccination campaign. However, the poor uptake of the COVID-19 vaccine among adolescents during the first phase likely restrained the MOH from extending the COVID-19 vaccination campaign to younger children under 12 years of age. The low coverage observed among adolescents placed an important barrier to controlling community transmission and protecting children from serious outcomes. Parents’ hesitancy may have been the reason behind this low coverage, driven by misinformation, rumours, and conspiracy theories about COVID-19 vaccines and mistrust towards health authorities [15].

Paediatricians’ recommendations have a positive impact on parents’ decisions on immunization in Palestine, as they are the most trusted advisors for parents [18]. Therefore, we aimed to assess the knowledge, attitudes, and practices (KAP) of paediatricians in the West Bank, Palestine, regarding COVID-19 vaccination for children younger than 12 years, to provide insights into the readiness of paediatricians to lead the paediatric COVID-19 vaccination campaign, identify potential barriers, and inform the development of targeted training programmes and communication strategies for paediatricians to improve COVID-19 vaccine uptake among children in Palestine.

## 2. Materials and Methods

### 2.1. Study Design and Setting

We conducted a cross-sectional study among paediatricians across the West Bank, Palestine. Data collection took place over a six-week period from 1 October to 8 November 2023.

### 2.2. Study Population and Sampling

The study population comprised all licensed paediatricians who were active members of the Paediatric Society-Palestine (PSP) and were practicing in either the public or private sector within the West Bank. We applied the following inclusion criteria: (i) licensed paediatricians who were registered with the PSP and (ii) practitioners who were actively practicing in the West Bank, Palestine. We applied the following exclusion criteria: (i) individuals who refused to provide verbal informed consent and (ii) paediatric residents, to ensure that the sample represented fully qualified practitioners whose recommendations carried substantial weight with parents. We invited all eligible paediatricians included in the PSP list to participate in the study to ensure maximum representativeness and statistical power.

### 2.3. Data Collection Tool and Procedure

We developed a structured questionnaire based on the review of relevant literature concerning healthcare professionals’ KAP regarding COVID-19 vaccination [19,20]. We designed the original tool in English, translated it into Arabic, and then back-translated it into English to ensure consistency and accuracy. The questionnaire included 45 items, with multiple-choice, closed-ended, semi-closed-ended, and open-ended questions. The questionnaire included the following sections: (i) Socio-demographic and professional characteristics (including data on age, gender, years of experience, primary sector of practice (public/private), governorate, and any previous formal training in vaccinology or infectious diseases), (ii) Personal vaccination and COVID-19 history (including the paediatrician’s own COVID-19 vaccination status, personal history of laboratory-confirmed COVID-19 infection, and the vaccination status of their children/grandchildren), (iii) Knowledge section containing eight questions assessing knowledge about COVID-19 vaccines for children (e.g., approved types, common side effects, age range recommended by Palestinian MOH, knowledge of safety and efficacy, and knowledge about safely administering the vaccine for children previously infected, and alongside childhood routine vaccinations), (iv) Attitudes section containing eleven questions, 6 of which asked about agreement with mandating COVID-19 vaccines for children, confidence in vaccine safety, factors associated with hesitancy in recommending vaccination, willingness to vaccinate their own children, and five 5-point Likert-scale questions (from “strongly disagree” to “strongly agree”) to gauge perceptions of vaccine safety, efficacy, and necessity for children, (v) Practice section consisting of 9 questions including respondents’ involvement in administering routine and COVID-19 vaccinations, how often they recommend parents to vaccinate eligible children, any parental concerns about routine vaccinations since the pandemic, trust in WHO and local health authorities recommendations in COVID-19 vaccination and any changes in their trust in health authorities.

Prior to the main study, we conducted a pilot study with five paediatricians (who were excluded from the final sample) to assess the clarity of the questionnaire, flow, and comprehensibility and to estimate the average time required for completion. We used feedback from this pilot to refine the survey instrument. Trained medical interns administered the survey via telephone. Interviewers entered all responses directly into the Kobo Toolbox form during the call to minimise data entry errors. We trained data collectors to administer the surveys in a neutral and non-judgemental manner to minimise information bias. Furthermore, the use of a closed-ended, structured questionnaire helped limit interviewer bias.

### 2.4. Data Analysis

We summarised quantitative variables as either means ± standard deviation or medians and ranges, based on their distribution. For qualitative variables, we estimated frequencies and percentages and used the chi-square test to examine associations; for cells with expected counts less than 5, we used Fisher’s exact test. We performed all analyses using the statistical software R (version 2025.05.1).

#### 2.4.1. Definition of the Outcome Variable

The primary outcome variable was paediatricians’ support for COVID-19 vaccination for children, derived from the survey question: “Do you agree with mandating vaccination of children under 12 years against COVID-19?” Respondents who selected “Yes, for all children” were categorized as supporting the mandate. All other responses (“No,” “Yes, but only for children at risk,” “No, for children over a certain age”) were categorized as not supporting the mandate.

#### 2.4.2. Calculation of KAP Scores

We calculated a knowledge score for each participant based on eight questions, yielding a range from 0 to 8 (one point per correct answer). We categorized scores as follows: 6–8 indicated good knowledge, 4–5 indicated moderate knowledge, and <4 indicated poor knowledge. We calculated an attitude score based on nine questions, yielding a range from 0 to 9. We excluded from the attitude score the survey question used to define the primary outcome (“Do you agree with mandating vaccination of children against COVID-19?”). We defined a positive attitude as a favourable and supportive mindset towards childhood COVID-19 vaccination, characterized by a belief in vaccination’s crucial role as a preventive measure a perception of its safety and efficacy. We categorized scores as follows: 7–9 indicated a positive attitude, 5–6 indicated a neutral attitude, and <5 indicated a negative attitude. We calculated the practice score based on 9 questions, yielding a range from 0 to 9. We categorized this score as follows: 7–9 indicated good practice, 5–6 indicated moderately good practice, and <5 indicated poor practice.

To justify these thresholds, we followed cut-off strategies commonly applied in similar KAP surveys among physicians in the region. Higher categories correspond to correct or favourable responses to at least 75–80% of items. Sensitivity analyses using alternative cut-offs (≥70% and ≥85%) showed no meaningful changes in category distributions or in the direction and significance of the main associations. Therefore, the original thresholds were retained for consistency with previous studies and to facilitate comparability.

#### 2.4.3. Bivariable and Multivariable Analysis

To identify factors associated with supporting COVID-19 vaccination for children under 12 years, we performed both bivariable and multivariable analyses. We first conducted a bivariable analysis to calculate crude prevalence ratios (PR). Subsequently, we built a multivariable Poisson regression model to calculate adjusted prevalence ratios (aPR), controlling for potential confounders. We included in the initial Poisson models all variables with a *p*-value of <0.20 in the bivariable analysis. We then employed a backward elimination approach, whereby variables were removed one by one based on the Akaike Information Criterion (AIC). We reported 95% confidence intervals for all PRs and considered a *p*-value of less than 0.05 to be statistically significant.

### 2.5. Ethical Considerations

We obtained ethical approval from the scientific research review board of the Public Health Directorate, Palestinian Ministry of Health (date: 26 September 2023; No.: 1392). We obtained oral informed consent from all participants after explaining the study’s objectives, the voluntary nature of participation, and the assurance of anonymity and confidentiality. We maintained strict confidentiality by not collecting any personally identifiable information alongside the survey data. The study complied with all relevant ethical guidelines, including the principles of the Declaration of Helsinki.

## 3. Results

### 3.1. Demographic Characteristics

Of the 367 eligible paediatricians, 323 (88%) participated in the study. Of all respondents, 69% were female. The median age was 51 years (range: 28–70), and 43% were aged between 40 and 59 years (Table 1). Of the participants, 56% were married, and 48% had children or grandchildren younger than 18 years. Work experience varied, with 36% practicing for 11–20 years and 25% for more than 20 years. Paediatricians were distributed across private (33%), public (34%), and both private and public sectors (33%); 36% worked in clinics, 35% in hospitals, and 28% in private practices. The most common specialties were general paediatrics (34%), pulmonology (11%), and cardiology (9.9%). Among respondents, 48% regularly attended continuing medical education (CME) activities, and 41% had received training on COVID-19 vaccination in children (Table 1).

### 3.2. Knowledge, Attitudes, and Practices

Among all respondents, 13% (n = 41) had a good knowledge score (≥6 correct answers out of 8 components); the mean knowledge score was 3.0 (SD = 2.1). Of all respondents, 31% correctly identified the WHO recommendation for vaccinating children, and 40% knew the Palestinian MOH recommended the COVID-19 vaccine for children; 37% were aware that the BNT162b2 (Pfizer-BioNTech) COVID-19 vaccine was authorized for children; 41% rated their self-assessed knowledge regarding vaccine safety and efficacy as high or very high, while 33% were aware that vaccination was safe for previously infected children; 66% could identify common side effects, mainly fever (32%), flu-like illness (21%), and local tenderness (18%) (Table 2).

Among respondents, 12% (n = 40) had a positive attitude score of (≥7 out of 9); the mean attitude score was 3.9 (SD = 2.4). Of respondents, 27% agreed that children of all ages should receive the vaccine, 25% agreed to mandating the vaccine for children at high risk (e.g., with asthma, immunodeficiency), and 20% agreed on vaccination of children 12 years and older; 30% were confident in vaccine safety, while 24% expressed hesitancy and 21% were very hesitant. The main concerns included safety (58%), lack of long-term data (56%), and parental hesitancy (55%). Only 17% of those with children or grandchildren were willing to vaccinate them; 46% and 41% agreed that vaccination reduces school absenteeism or protects against long-term effects, respectively (Table 2).

Of all respondents, 8.4% (n  =  27) had a good practice score of (≥7 out of 9), with the mean practice score being 3.98 (SD = 1.7). Only 34% were directly involved in administering childhood vaccines, and 50% in COVID-19 vaccination. Twenty-two percent reported always suggesting the COVID-19 vaccine for children in their practices, while 27% never did. Trust in COVID-19 vaccination guidance was modest, with 54% expressing little or no trust in MoH or WHO recommendations (Table 2). Thirty-seven percent of respondents reported an increase in parental concerns toward routine vaccine safety and efficacy, and 33% of participants had a decreased level of trust in health authorities’ COVID-19 recommendations since the pandemic (Table S4 ). Of all respondents, 11% (n  =  36) had a good total KAP score of ≥19 out of 26, with the mean total KAP score being 10.9 (SD  =  4.8).

Knowledge and attitude scores were strongly correlated (r = 0.83, *p* < 0.001). However, correlations between knowledge and practice (r = 0.086, *p* = 0.121) and attitude and practice (r = 0.076, *p* = 0.175) were weak and not statistically significant (Table 3).

In the univariable analysis, sociodemographic factors such as gender, age, marital status, years of experience, and specialty were not significantly associated with support for COVID-19 vaccination of children. However, the proportion of paediatricians recommending COVID-19 vaccination for children was significantly higher among those attending CME (PR 3.11, 95% CI 2.02–4.80, *p* < 0.001) and receiving training on COVID-19 vaccination (PR 2.74, 95% CI 1.86–4.02, *p* < 0.001) (Table 4).

In terms of knowledge, those aware of BNT162b2 authorization (PR 3.01, 95% CI 1.90 to −4.76) and those who self-rated their vaccine efficacy and safety knowledge as very high (PR 2.42, 95% CI 1.22–4.76, *p* < 0.001) were significantly more likely to support mandating COVID-19 vaccination for children (Table 4).

In terms of attitudes, paediatricians who were very confident in vaccine safety were more likely to support mandating COVID-19 vaccination for children compared with hesitant respondents (PR 8.50, 95% CI 4.44–16.3). Agreement that vaccination reduces transmission to families (PR 2.64, 95% CI 1.51–4.63) and prevents long-term complications (PR 1.99, 95% CI 1.05–3.75) was a significant predictor of supporting COVID-19 vaccination for children (Table 4).

Paediatricians who “always” advised the COVID-19 vaccine for eligible children in their practice were more likely to support the COVID-19 vaccine for children (64% vs. 11–25% in other groups). Trust in MOH (PR 4.45, 95% CI 2.67–7.42) and WHO (PR 7.14, 95% CI 3.58–14.2) was also associated with supporting the COVID-19 vaccine for children (Table 4).

Ninety percent of paediatricians with an overall good knowledge score, compared with 16% of those with a poor knowledge score, supported COVID-19 vaccination for children of all ages (PR 5.62, CI: 4.07–7.78, *p* < 0.001). Similarly, 93% of respondents with positive attitudes recommended COVID-19 vaccination compared with 13% with negative attitudes (PR 7.40, CI: 5.17–10.6, *p* < 0.001). 40% of those with good practices (PR 2.27, CI: 1.37–3.77, *p* = 0.006) were more likely to recommend COVID-19 vaccination for children. Of respondents with a good total KAP score (n = 36), 100% had supported COVID-19 vaccination for children, whereas 17% of those with a poor KAP score (n = 287) supported the same (PR 5.74, CI: 4.50–7.40, *p* < 001) (Table 4).

In the final multivariable Poisson regression model, four factors were independently and significantly associated with supporting COVID-19 vaccination for children under 12 years after adjusting for potential confounders. Paediatricians who regularly attended CME were more likely to support the mandate compared to those who did not (aPR = 1.43, 95% CI: 1.01–2.56, *p*-value = 0.045). Similarly, greater trust in WHO recommendations (aPR = 3.14, 95% CI: 1.39–7.78, *p* value = 0.047), positive attitude score (aPR = 1.26, 95% CI: 0.36–4.37, *p* value = 0.041), and good total KAP score (aPR = 1.10, 95% CI: 1.00–1.21, *p* value = 0.044) were also significant independent predictors. Other variables, including gender, age, and receiving training on COVID-19 vaccination, did not remain statistically significant after adjustments (Table 4).

## 4. Discussion

Our study indicated a high level of hesitancy among paediatricians in the West Bank, Palestine, towards COVID-19 vaccination for children under 12 years. Few respondents had good knowledge, positive attitudes, or good practices towards childhood COVID-19 vaccination, with many expressing concerns about safety, efficacy, and potential long-term effects. Participation in CME activities, receipt of formal training on COVID-19 vaccination in children, trust in local health authorities and WHO recommendations, and a higher overall total KAP score were factors associated with supporting COVID-19 vaccination for children.

In this study, only about one quarter (≈27%) of paediatricians supported COVID-19 vaccination for children under 12 years; a large proportion were hesitant, with only 11% reporting high confidence in COVID-19 vaccination for children. Safety, efficacy of the vaccines, lack of sufficient data on COVID-19 vaccine long-term effects on children, and parental hesitancy were the most common factors cited by Palestinian paediatricians. The low support for the COVID-19 vaccination for children among Palestinian paediatricians reflects a fundamental gap in knowledge regarding the established safety and efficacy evidence of administering COVID-19 vaccines in children. This was reflected in the low mean knowledge score observed among Palestinian paediatricians. However, this hesitancy does not align with the strong scientific evidence supporting paediatric COVID-19 vaccination. Randomized trials reported that the BNT162b2 vaccine is safe and produces a strong immune response in children 5–11 years, and the appropriately lower-dose regimens are immunogenic even in younger age groups (6 months–4 years) [7,21]. Multiple systematic reviews and large clinical trials indicated that COVID-19 vaccines are generally safe for children and adolescents, with most adverse events being mild to moderate (e.g., injection site pain, fever, headache, fatigue), with serious adverse events being rare and typically unrelated to vaccination. Myocarditis and pericarditis are rare, transient, and resolve fully in most cases [22,23,24]. Studies in real-world settings also indicated that the vaccine protects children well against severe illness and offers some protection against infection, though its effectiveness has varied with different virus variants [25].

The Palestinian Expanded Programme on Immunization (EPI) is widely recognized as a public health achievement, especially given the region’s political and economic challenges. High vaccination coverage proportions (97–99%) of key vaccines, rapid introduction of new vaccines, and significant reductions in vaccine-preventable diseases highlighted the programme’s effectiveness [26,27]. The low support and hesitancy towards paediatric COVID-19 vaccine by paediatricians observed in our study could impact the EPI in Palestine and could pose a threat to this successful, vital programme that has historically protected generations of Palestinian children. Evidence from other countries shows that hesitancy around COVID-19 vaccination can spill over to routine immunization, leading to decreased coverage and increased risk of outbreaks of vaccine-preventable diseases [28,29]. The low support of paediatric COVID-19 vaccination observed in our study is consistent with other studies reporting substantial heterogeneity in healthcare provider recommendation behaviour during the pandemic. Several KAP surveys of paediatricians and family physicians reported variable recommendation rates and important knowledge gaps linked to concerns about long-term safety and local guidance [30,31]. In the WHO EMRO region, and specifically in Tunisia, only 41% of paediatricians were willing to recommend COVID-19 vaccination for children, and most would do so only for children with chronic diseases [32]. This suggests that low recommendation rates are not unique to our setting and are often driven by similar challenges. Multiple contextual factors in Palestine likely influenced the observed levels of support for COVID-19 vaccination among paediatricians. Unequal vaccine supply compared to countries like Israel, cold-chain disruptions, transport delays, and staff shortages have hindered vaccine delivery and created uncertainty among healthcare providers and parents. Political restrictions, including procurement policies, movement limitations, and perceived coercive mandates, have further undermined public trust and access. Long-standing mistrust in health authorities—fuelled by misinformation, inconsistent messaging, and concerns over rapid vaccine development or political influence—has also contributed to hesitancy, even among parents who otherwise accept routine childhood immunizations. Collectively, these supply, logistical, political, and trust-related barriers provide additional context to the low levels of support observed in this study and highlight the limitations in generalizing these findings to other regions.

We observed a strong correlation between knowledge and attitude towards childhood COVID-19 vaccination, but weak correlations between either knowledge or attitude and reported practice. Those findings suggest that improving knowledge may change attitudes, but changing actual behaviour may require additional training in communication skills, institutional protocols that empower clinicians’ recommendations, and interventions that rebuild trust. In other vaccine contexts (e.g., HPV, influenza), studies have repeatedly indicated that a clinician’s explicit recommendation is one of the single most powerful determinants of parent uptake, and changing provider behaviours can increase vaccination rates [33,34]. We found that paediatricians who always advise parents to have their children vaccinated against COVID-19 were much more likely to support vaccination for children under 12 years, regardless of age group or presence of underlying medical conditions, suggesting that good knowledge and positive attitudes need to be turned into consistent routine practice, rather than just occasional advice.

In our study, continuous education of paediatricians and their formal training were associated with higher recommendation rates. Thus, educating paediatricians on vaccine-preventable diseases and updated recommendations, vaccine safety, co-administration, and local MOH guidance could improve both their attitudes and readiness to recommend the vaccine. Secondly, knowledge alone is not enough; communication skills and building trust are also essential. Paediatricians in our study often mentioned parental hesitancy as a barrier. Half of the respondents perceived an increase in parental concerns towards routine childhood vaccines, underscoring the continuing challenge of parental hesitancy in the post-pandemic era. Meta-analyses and umbrella reviews indicated sizable parental hesitancy rates globally (pooled hesitancy estimates often in the ~30–40% range in some periods), with safety and long-term outcomes cited most commonly [34,35]. Paediatricians who completed structured COVID-19 vaccine curricula had a marked increase in knowledge (median scores rose from 79% to 93%) and sustained improvements in confidence when discussing vaccines with families.

### Limitations

This study had several limitations. Firstly, the cross-sectional design captured data at a single point in time, which prevented the establishment of causal relationships between vaccination recommendation rates and associated factors. Secondly, the study was susceptible to non-response bias; paediatricians with strong opinions, either highly in favour of or against vaccination, may have been more likely to participate, potentially skewing the results. However, we have achieved a high response rate in this study, minimising the effects of low response. Thirdly, the reliance on self-reported practices, such as vaccine recommendation frequency, may have been subject to social desirability bias, where respondents might have provided answers they perceived to be more socially acceptable rather than their actual practices. We trained the interviewers and piloted the questionnaire to minimise this potential bias. Finally, in November 2023, the WHO issued a recommendation decreasing the priority for vaccinating children against COVID-19. This could influence some of our respondents’ perceptions of the benefit of vaccinating individual children. Although we could not mitigate the impact of this unexpected development, the recommendation was announced when the data collection was mostly completed.

## 5. Conclusions

In conclusion, this study identified low COVID-19 vaccination recommendation rates among paediatricians in the West Bank, with only a quarter routinely advising COVID-19 vaccination for children under 12 years, driven by substantial knowledge deficits and safety concerns. The strong correlation between good knowledge and positive attitudes underscores that enhancing evidence-based education through targeted CME and formal training is a critical first step to increase the number of paediatricians who recommend COVID-19 vaccination to children. However, bridging the gap between positive intention and consistent practice requires a dual approach: equipping paediatricians with advanced communication skills and institutional support to confidently address parental hesitancy while concurrently implementing public health campaigns to rebuild community trust and disseminate clear safety information, thereby fostering a more supportive environment for childhood vaccination. The WHO updated its recommendations on 10 November 2023, recommending a single-dose vaccine primarily for children under 12 years with comorbidities or at higher risk of severe disease. Despite the changing recommendations, our findings provided evidence on paediatricians’ perspectives on the current recommendation status during an ongoing public health emergency. Strengthening paediatricians’ role in evidence-based communication and vaccine advocacy remains essential. Beyond the context of COVID-19, well-informed and confident paediatricians play a critical role in guiding parents, countering misinformation, and promoting timely vaccination during future outbreaks or pandemics. Their engagement ensures that public health recommendations are effectively translated into practice, improves vaccine confidence among communities, and strengthens overall health system preparedness for emerging infectious diseases. Investing in ongoing training, communication skills, and supportive institutional frameworks equips paediatricians to respond rapidly and effectively to both routine immunization challenges and novel infectious threats.

## 6. Recommendations

Our data indicate two high-impact action areas: (1) rapid, practical CME and training that updates paediatricians on current evidence, age indications, and co-administration safety; and (2) provider communication training and system-level supports (reminders, audit and feedback, clear MOH guidance) to convert positive knowledge and attitudes into routine, presumptive recommendations. Finally, future research should evaluate the effect of targeted CME and communication skill interventions in increasing both paediatrician recommendation behaviour and actual vaccination uptake among children in Palestine. Implementation studies that measure both provider behaviour change and downstream parental uptake will be essential to move from improved knowledge and attitudes to measurable public health impact.

## Figures and Tables

**Table 1:** Socio-demographic characteristics in the survey; knowledge, attitude, and practices of paediatricians in the West Bank, Palestine, regarding COVID-19 vaccination among children younger than 12 years, 2023, N = 323.

Characteristics		N	%
Gender	Female	222	69
	Male	101	31
Age group	≥40 <60	140	43
	≥60	95	30
	<40	88	27
Marital status	Married	181	56
	Single	80	25
	Divorced	56	17
	Widowed	6	1.9
Work experience	11–20 years	117	36
	>20 years	82	25
	<5 years	64	20
	5–10 years	60	19
Primary specialty	General Paediatrics	109	34
	Paediatric Pulmonology	36	11
	Paediatric Cardiology	32	9.9
	Paediatric Surgery	30	9.3
	Paediatric Oncology	27	8.4
	Paediatric Infectious Diseases	24	7.4
	Paediatric Endocrinology	23	7.1
	Paediatric Neurology	21	6.5
	Paediatric Allergy	17	5.2
	Other	4	1.2
Primary place of practice	Public sector	110	34
	Private sector	108	33
	Both sectors	105	33
Regular CME * attendance	No	167	52
	Yes	156	48
Formal training on COVID-19 vaccination	Yes	131	41
	No	192	59
History of COVID-19 infection	Yes	164	51
	No	159	49
History of COVID-19 infection	Yes	164	51
	No	159	49
COVID-19 vaccination status	Fully vaccinated	244	76
	One dose	55	17
	Not vaccinated	24	7
Willingness for regular vaccination	Willing	155	48
	Unsure/Depends	138	43
	Not Willing	30	9
Underlying medical conditions	Yes	127	39
	No	115	36
	Prefer not to say	81	25
Children/Grandchildren <18	No	167	52
	Yes	156	48
Children/Grandchildren infected with COVID-19	Does not have children	167	52
	Yes	124	38
	No	32	10
Children/Grandchildren vaccinated for COVID-19	Does not have children	167	52
	No	128	40
	Yes	28	8

* Continuing medical education.

**Table 2:** Correct knowledge, positive attitudes, and good practices for COVID-19 vaccination among paediatricians, West Bank, Palestine, 2023, N = 323.

Domain	Question	N	%
Knowledge	Knowledge of Common Side Effects of COVID-19 Vaccination in Children	214	66
	Knowledge Rating about Safety and Efficacy of COVID-19 Vaccines for Children	133	41
	Knowledge of the Palestinian Ministry of Health Recommendations	129	40
	Knowledge of Safety of Co-administering COVID-19 Vaccine with Other Childhood Vaccines	123	38
	COVID-19 Vaccines Authorized for Children in the West Bank, Palestine	120	37
	Knowledge of Vaccination for Previously Infected Children	108	33
	Knowledge of the WHO Recommendation for Vaccinating Children	100	31
	Knowledge of COVID-19 Vaccines Recommended by WHO	44	14
Attitudes	COVID-19 generally does not cause severe illness among children.	226	70
	Administering COVID-19 vaccines to children may help decrease the number of days they miss school.	147	46
	Vaccinating children is effective in reducing the severity of COVID-19 if they are infected with the virus	136	42
	Vaccinating children against COVID-19 may lower their risk of transmitting the virus to their family members.	131	41
	Childhood COVID-19 vaccination offers protection against long-term health effects often associated with the virus (long COVID).	133	41
	Vaccinating children is effective in reducing their chances of contracting COVID-19.	131	41
	COVID-19 may lead to severe illness or even death in children.	130	40
	Confidence in Safety of Vaccines for Children	98	30
	Willingness to Vaccinate Own Children against COVID-19	55	17
Practices	Change in trust in Health Authorities regarding COVID-19 after the pandemic	217	67
	Changes in Parental Concerns About Vaccine Safety and/or Effectiveness since Pandemic	180	56
	Frequency of Recommending COVID-19 Vaccination for Eligible Children	166	51
	Involvement in the Administration of COVID-19 Vaccines to Children	160	50
	Trust in WHO Recommendations on COVID-19 Vaccination	149	46
	Trust in Health Authorities on COVID-19 Vaccination	147	46
	Involvement in Routine Vaccinations for Children	110	34
	Level of Experience in Administering Routine Vaccinations to Children	81	25
	Number of Children Vaccinated Monthly	78	24
Outcome	Supporting vaccination for children under 12 years against COVID-19	86	27

**Table 3:** Correlation between knowledge, attitudes, and practices, survey of COVID-19 vaccination among paediatricians, West Bank, Palestine, 2023.

Variables	Correlation Coefficient (r)	*p*
Knowledge Score vs. Attitude Score	0.825	<0.001
Knowledge Score vs. Practice Score	0.086	0.121
Attitude Score vs. Practice Score	0.076	0.175

**Table 4:** Determinants of supporting COVID-19 vaccination for children under 12 years by selected characteristics, West Bank, Palestine, 2023.

Characteristic	Category	Support COVID-19 Vaccination for Children Under 12 Years		Crude Prevalence Ratios (95% CI)	*p*	Adjusted Prevalence Ratios (95% CI)	*p*
		n /N	%				
Gender	Female	54/222	24	ref	0.1762	-	-
	Male	32/101	32	1.3 (0.90–1.88)		-	-
Age group (years)	<40	23/88	26	ref	0.7337	-	-
	40–60	35/140	25	0.96 (0.61–1.51)		-	-
	>60	28/95	29	1.13 (0.71–1.80)		-	-
Attend CME regularly	No	22/167	13	ref	<0.001	ref	0.045
	Yes	64/156	41	3.11 (2.02–4.80)		1.43 (1.01–2.56)	
Received training on COVID-19 vaccination	No	30/192	16	ref	<0.001	-	-
	Yes	56/131	43	2.74 (1.86–4.02)		-	-
Does the WHO recommend COVID-19 vaccination for children below 12 years old?	No	19/119	16	ref	<0.001	-	-
	Yes	48/100	48	3.01 (1.90–4.76)		-	-
	I don’t know	19/104	18	1.14 (0.64–2.04)		-	-
How would you rate your knowledge about the efficacy and safety of the COVID-19 vaccine?	Very low	9/60	15	ref	<0.001	-	-
	Low	9/85	11	0.71 (0.29–1.67)		-	-
	Moderate	10/45	22	1.48 (0.66–3.34)		-	-
	High	33/64	52	3.43 (1.79–6.56)		-	-
	Very high	25/69	36	2.42 (1.22–4.76)		-	-
How confident are you in the safety of COVID-19 vaccines for children?	Very hesitant	8/68	12	ref	<0.001	-	-
	Hesitant	12/78	15	1.31 (0.57–3.01)		-	-
	Neutral	19/79	24	2.05 (0.96–4.37)		-	-
	Somewhat confident	11/62	18	1.51 (0.65–3.51)		-	-
	Very confident	36/36	100	8.50 (4.44–16.3)		-	-
Vaccinating children against COVID-19 may lower their risk of transmitting the virus to their family members	Strongly disagree	11/52	21	ref	<0.001	-	-
	Disagree	2/36	5.6	0.26 (0.06–1.12)		-	-
	Neither agree nor disagree	11/57	19	0.92 (0.44–1.92)		-	-
	Agree	14/54	26	1.23 (0.62–2.45)		-	-
	Strongly agree	43/77	56	2.64 (1.51–4.63)		-	-
	I don’t know	5/47	11	0.51 (0.19–1.34)		-	-
Childhood COVID-19 vaccination offers protection against long-term health effects associated with the virus	Strongly disagree	9/39	23	ref	<0.001	-	-
	Disagree	11/59	19	0.81 (0.37–1.80)		-	-
	Neither agree nor disagree	6/49	12	0.53 (0.21–1.36)		-	-
	Agree	28/72	39	1.69 (0.89–3.20)		-	-
	Strongly agree	28/61	46	1.99 (1.05–3.75)		-	-
	I don’t know	4/43	9.3	0.40 (0.13–1.21)		-	-
How frequently do you advise the COVID-19 vaccination for children in your practice?	Never	13/87	15	ref	<0.001	-	-
	Rarely	8/70	11	0.76 (0.34–1.74)		-	-
	Occasionally	8/48	17	1.12 (0.49–2.51)		-	-
	Often	12/48	25	1.67 (0.83–3.37)		-	-
	Always	45/70	64	4.30 (2.53–7.31)		-	-
Do you trust the health authorities’ recommendations on COVID-19 vaccination?	A little	15/114	13	ref	<0.001	-	-
	Moderately	20/77	26	1.97 (1.08–3.61)		-	-
	A lot	41/70	59	4.45 (2.67–7.42)		-	-
	Not at all	10/62	16	1.23 (0.59–2.56)		-	-
Do you trust the WHO recommendations on COVID-19 vaccination?	A little	8/97	8.2	ref	<0.001	ref	0.047
	Moderately	20/76	26	3.19 (1.49–6.84)		2.13 (0.93–5.31)	
	A lot	43/73	59	7.14 (3.58–14.2)		3.14 (1.39–7.78)	
	Not at all	15/77	20	2.36 (1.06–5.28)		2.04 (0.88–5.09)	
Attitude score	Negative	28/224	13	ref	<0.001	ref	0.041
	Neutral	21/59	36	2.84 (1.74–4.63)		2.15 (1.13–4.03)	
	Positive	37/40	93	7.40 (5.17–10.6)		1.26 (0.36–4.37)	
Total KAP score	Poor	50/287	17	ref	<0.001	ref	0.044
	Good	36/36	100	5.74 (4.50–7.40)		1.10 (1.00–1.21)	

n = number of respondents in the category who support COVID-19 vaccination for children under 12 years; N = total number of respondents in the category; ref = reference category used in regression analysis.

## Data Availability

The data presented in this study are available on request from the corresponding author. The data are not publicly available due to privacy.

## Supplementary Materials

The following supporting information can be downloaded at: https://www.mdpi.com/article/10.3390/vaccines13121236/s1, Table S1: Knowledge About COVID-19 Vaccination in Children, Knowledge, Attitudes and Practices survey of COVID-19 vaccination among paediatricians, West Bank, Palestine, 2023; Table S2: Attitudes Towards COVID-19 in Children, Knowledge, Attitudes and Practices survey of COVID-19 vaccination among paediatricians, West Bank, Palestine, 2023; Table S3: Attitudes Towards COVID-19 in Children, Knowledge, Attitudes and Practices survey of COVID-19 vaccination among paediatricians, West Bank, Palestine, 2023; Table S4: Practices and Trust Related to COVID-19 Vaccination in Children, Knowledge, Attitudes and Practices survey of COVID-19 vaccination among paediatricians, West Bank, Palestine, 2023; Table S5: Participant’s sociodemographic data about recommending COVID-19 vaccination for children; Table S6: Knowledge factors influenced paediatricians recommending COVID-19 vaccine for children; Table S7: Attitude factors influenced paediatricians recommending COVID-19 vaccine for children; Table S8: Practice factors influenced paediatricians recommending COVID-19 vaccine for children; Informed Consent sheet; Questionnaire.
