# Supplementary material for: Knowledge, Attitude, and Practices of Paediatricians in the West Bank, Palestine, Regarding COVID-19 Vaccination Among Children Younger than 12 Years: A Cross-Sectional Study, October to November 2023
Source: Vaccines (Basel). 2025 Dec 11;13(12):1236. doi: 10.3390/vaccines13121236 (PMC12737588; doi:10.3390/vaccines13121236)
Supplement: Supplementary file 1 [file vaccines-13-01236-s001.zip › vaccines-3964826-supplementary.pdf]

Supplementary materials

Table S1: Knowledge About COVID-19 Vaccination in Children, Knowledge, Attitudes and Practices survey of COVID-19 vaccination among paediatricians, West Bank, Palestine, 2023

Knowledge Variable	Response	Frequency (n)	Percentage (%)
Knowledge of WHO Recommendation for Vaccinating Children	Yes	100	31
	No	119	37
	I don't know	104	32
Knowledge of COVID-19 Vaccines Recommended by WHO	Pfizer only	44	14
	Others	279	86
Knowledge of Palestinian Ministry of Health Recommendations	12 years and above	129	40
	5 years and above	82	25
	Not aware	112	35
COVID-19 Vaccines Authorized for Children in West Bank, Palestine	Pfizer	120	37
	I don't know	113	35
	Other	90	28
Knowledge Rating about Safety and Efficacy of COVID-19 Vaccines for Children	Very Low	60	19
	Low	85	26
	Moderate	45	14
	High	64	20
	Very High	69	21
Knowledge of Common Side Effects of COVID-19 Vaccination in Children	fever	104	32
	Flu-like illness	69	21
	Tenderness at injection site	57	18
	pericarditis	49	15
	I don't know	44	14
	Post COVID syndrome	43	13
	fatigue	41	13
	Myalgia	41	13
	No side effects	39	12
	headache	1	0.3
	hypoactivity	1	0.3
	Multiple inflammatory systemic syndrome	1	0.3

**Knowledge Variable**	**Response**	**Frequency (n)**	**Percentage (%)**
Knowledge of Vaccination for Previously Infected Children	Yes	108	33
	No	97	30
	I don't know	118	37
Knowledge of Safety of Co-administering COVID-19 Vaccine with Other Childhood Vaccines	Yes	123	38
	No	82	25
	I don't know	118	37

Table S2: Attitudes Towards COVID-19 in Children, Knowledge, Attitudes and Practices survey of COVID-19 vaccination among paediatricians, West Bank, Palestine, 2023

Attitude Variable	Response	Frequency (n)	Percentage (%)
Supporting COVID-19 Vaccination For Children <12 years old (main outcome)	No	90	28
	Yes, for all	86	27
	Yes, for children at risk	81	25
	Yes, children over 12 of age	66	20
Confidence in Safety of Vaccines for Children	hesitant	78	24
	neutral	79	24
	Somewhat confident	62	19
	Very confident	36	11
	Very hesitant	68	21
Factors Contributing to Hesitancy About Recommending COVID-19 Vaccines for Children	Safety concerns	187	58
	Efficacy concerns	166	51
	Lack of data...	181	56
	Parental vaccine...	177	55
	Not ant hesitant	36	11
Willingness to Vaccinate Own Children Against COVID-19 (if applicable)	Yes, I would...	55	17
	No, I would not...	52	16
	I do not have...	166	51
	Prefer not to say	50	15

Table S3: Attitudes Towards COVID-19 in Children, Knowledge, Attitudes and Practices survey of COVID-19 vaccination among paediatricians, West Bank, Palestine, 2023

Statement	Agree % (n)	Disagree % (n)	I Don't Know % (n)	Neither Agree nor Disagree % (n)	Strongly Agree % (n)	Strongly Disagree % (n)
Vaccinating children against COVID-19 may lower their risk of transmitting the virus to their family members.	17% (54)	11% (36)	15% (47)	18% (57)	24% (77)	16% (52)
Childhood COVID-19 vaccination offers protection against long-term health effects often associated with the virus (long COVID).	22% (72)	18% (59)	13% (43)	15% (49)	19% (61)	12% (39)
COVID-19 generally does not cause severe illness among children.	15% (48)	17% (54)	14% (46)	17% (54)	15% (49)	22% (72)
Administering COVID-19 vaccines to children may help decrease the number of days they miss school.	27% (88)	13% (43)	15% (48)	11% (34)	18% (59)	16% (51)
COVID-19 may lead to severe illness or even death in children.	25% (81)	15% (49)	15% (47)	14% (44)	15% (49)	16% (53)
Vaccinating children is effective in reducing the severity of COVID-19 if they infected with the virus	21% (69)	13% (41)	14% (46)	18% (57)	21% (67)	13% (43)
Vaccinating children is effective in reducing their chances of getting COVID-19.	20% (63)	15% (47)	15% (47)	15% (49)	21% (68)	15% (49)

Table S4: Practices and Trust Related to COVID-19 Vaccination in Children, Knowledge, Attitudes and Practices survey of COVID-19 vaccination among paediatricians, West Bank, Palestine, 2023

Practice Variable	Response	Frequency (n)	Percentage (%)
Involvement in Routine Vaccinations for Children	Yes, I am directly...	110	34
	No, I am not directly, but oversees	92	28
	No, I am not involved...	121	37
Number of Children Vaccinated Monthly	Less than 10 children	32	9.9
	10-20 children	24	7.4
	21-50 children	28	8.7
	More than 50 children	26	8
	Don't vaccine	213	66
Level of Experience in Administering Routine Vaccinations to Children	Very Experienced	26	8
	Experienced	20	6.2
	Somewhat Experienced	35	11
	Limited Experience	32	9.9
	No Experience	210	65
Involvement in Administration of COVID-19 Vaccines to Children	Yes, I administer...	1	0.3
	Yes, I oversee vaccine...	159	49
	No, I am not involved...	163	50
Frequency of Recommending COVID-19 Vaccination for Eligible Children	Always	70	22
	Often	48	15
	Occasionally	48	15
	Rarely	70	22
	Never	87	27
Changes in Parental Concern About Vaccine Safety and/or Effectiveness Since Pandemic	It has increased a lot	82	25
	It has increased slightly	61	19

**Practice Variable**	**Response**	**Frequency (n)**	**Percentage (%)**
	No, it has not changed	66	20
	It has decreased slightly	55	17
	It has decreased a lot	59	18
Trust in Health Authorities on COVID-19 Vaccination	Not at all	62	19
	A little	114	35
	Moderately	77	24
	A lot	70	22
Trust in WHO Recommendations on COVID-19 Vaccination	Not at all	77	24
	A little	97	30
	Moderately	76	24
	A lot	73	23
Change in Trust in Health Authorities Regarding COVID-19 After the Pandemic	Decreased	106	33
	Remained unchanged	114	35
	Increased	103	32

Table S5: Participant's sociodemographic data about recommending COVID-19 vaccination for children

Category	n/N	Prevalence (%)	Prevalence ratio (95% CI)	P value
**Gender**				
Female	54/222	24	ref	0.1762
Male	32/101	32	1.3 (0.9 - 1.88)	
**Age**				
<40	23/88	26	ref	0.7337
<60	35/140	25	0.96 (0.61 - 1.51)	
>60	28/95	30	1.13 (0.71 - 1.8)	
**Marital Status**				
Divorced	15/56	27	ref	0.8017
Married	46/181	25	0.95 (0.58 - 1.56)	
Single	24/80	30	1.12 (0.65 - 1.94)	
Widowed	1/6	17	0.62 (0.1 - 3.92)	
**Have children/grandchildren <18 years old**				
No	45/167	27	ref	0.9005
Yes	41/156	26	0.98 (0.68 - 1.4)	
**Years of Experience**				
<5	15/64	23	ref	0.2907
>20	21/82	26	1.09 (0.61 - 1.94)	
11-20	38/117	33	1.39 (0.83 - 2.32)	
5-10	12/60	20	0.85 (0.44 - 1.67)	
**Practice Place**				
private and public sectors	30/105	29	ref	0.6806
Private sector	30/108	28	0.97 (0.63 - 1.49)	
Public sector	26/110	24	0.83 (0.53 - 1.3)	
**Healthcare facility type**				
Clinic	32/117	27	ref	0.8275
Hospital	28/114	25	0.9 (0.58 - 1.39)	
Private practice	26/92	28	1.03 (0.67 - 1.6)	
**Primary Speciality**				

General Paediatrics	29/109	27	ref	0.6528
Paediatric Allergy and Immunology	6/17	35	1.33 (0.65 - 2.71)	
Paediatric Cardiology	8/32	25	0.94 (0.48 - 1.85)	
Paediatric Endocrinology	7/23	30	1.14 (0.57 - 2.29)	
Paediatric Infectious Diseases	7/24	29	1.1 (0.55 - 2.2)	
Paediatric Neurology	7/21	33	1.25 (0.63 - 2.47)	
Paediatric Oncology	4/27	15	0.56 (0.21 - 1.45)	
Paediatric Pulmonology	9/36	25	0.94 (0.49 - 1.79)	
Paediatric Surgery	7/30	23	0.88 (0.43 - 1.8)	
Paediatric Gastroenterology	0/1	0	0 (0 - NaN)	
Paediatric ICU	1/1	100	3.76 (2.75 - 5.13)	
Paediatric Neonatology	0/1	0	0 (0 - NaN)	
Paediatric Nephrology	1/1	100	3.76 (2.75 - 5.13)	
**Attend CME regularly**				
No	22/167	13	ref	<0.0001**
Yes	64/156	41	3.11 (2.02 - 4.8)	
**Previously infected with COVID-19**				
No	44/159	28	ref	0.7068
Yes	42/164	26	0.93 (0.64 - 1.33)	
**Received COVID-19 Vaccine**				
No	6/24	25	ref	0.8402
Yes, one dose	13/55	24	0.95 (0.41 - 2.19)	
Yes, fully vaccinated	67/244	28	1.1 (0.53 - 2.26)	
**Willing to receive COVID-19 regularly**				
No	6/30	20	ref	0.0157*
Unsure/Depends on the recommendations	48/138	35	1.74 (0.82 - 3.69)	
Yes	32/155	21	1.03 (0.47 - 2.25)	
**Underlying medical conditions**				
No	28/115	24	ref	0.7012
Prefer not to say	24/81	30	1.22 (0.76 - 1.94)	
Yes	34/127	27	1.1 (0.71 - 1.69)	
**Has Children/Grandchildren**				

**previously infected with COVID-19?**				
No	10/32	31	ref	0.7781
I don't have children/grandchildren	45/167	27	0.86 (0.49 - 1.53)	
Yes	31/124	25	0.8 (0.44 - 1.45)	
**Has Children/Grandchildren received COVID-19 vaccine?**				
No	32/128	25	ref	0.7409
I don't have children/grandchildren	45/167	27	1.08 (0.73 - 1.59)	
Yes	9/28	32	1.29 (0.69 - 2.38)	
**Received training on COVID-19 vaccination**				
No	30/192	16	ref	<0.0001**
Yes	56/131	43	2.74 (1.86 - 4.02)	

Table S6: Knowledge factors influenced paediatricians recommending COVID-19 vaccine for children

Category	n/N	Prevalence (%)	Prevalence ratio (95% CI)	P value
**Are you aware of WHO COVID-19 vaccination for children?**				
I don't know	19/104	18	ref	<0.0001
No	19/119	16	0.87 (0.49 - 1.56)	
Yes	48/100	48	2.63 (1.67 - 4.14)	
**Which COVID-19 vaccines authorized by WHO for children?**				
other	50/279	18	ref	<0.0001
Pfizer	36/44	82	4.57 (3.43 - 6.08)	
**At What Age Does the Palestinian MOH Recommend COVID-19 Vaccination for Children**				
12 years and above	55/129	43	ref	<0.0001
5 years and above	16/82	20	0.46 (0.28 - 0.74)	
Not aware	15/112	13	0.31 (0.19 - 0.52)	
**Which COVID-19 Vaccines Authorized for Children in West Bank, Palestine?**				
I don't know	16/113	14	ref	<0.0001
Moderna	18/89	20	1.43 (0.77 - 2.64)	
Pfizer	52/120	43	3.06 (1.86 - 5.04)	
Sputnik	0/1	0	0 (0 - NaN)	
**How would you rate your knowledge about efficacy and safety of COVID-19 vaccine?**				
high	33/64	52	ref	<0.0001
low	9/85	11	0.21 (0.11 - 0.4)	
moderate	10/45	22	0.43 (0.24 - 0.78)	
very high	25/69	36	0.7 (0.47 - 1.04)	
very low	9/60	15	0.29 (0.15 - 0.56)	
**COVID-19 vaccine can be given to children previously infected with the virus**				
I don't know	21/118	18	ref	<0.001
No	16/97	17	0.93 (0.51 - 1.68)	
Yes	49/108	45	2.55 (1.64 - 3.96)	

**COVID-19 vaccine is safe to administer with other childhood vaccines**				
I don't know	18/118	15	ref	<0.001
No	13/82	16	1.04 (0.54 - 2)	
Yes	55/123	45	2.93 (1.83 - 4.68)	

Table S7: Attitude factors influenced paediatricians recommending COVID-19 vaccine for children

Category	n/N	Prevalence (%)	Prevalence ratio (95% CI)	P value
**How confident are you in the safety of COVID-19 vaccines for children?**				
hesitant	12/78	15	ref	<0.001
neutral	19/79	24	1.56 (0.81 - 3)	
somewhat confident	11/62	18	1.15 (0.55 - 2.43)	
very confident	36/36	100	6.5 (3.86 - 10.94)	
very hesitant	8/68	12	0.76 (0.33 - 1.76)	
**Would you give COVID-19 vaccine to your child/grandchild in the future?**				
I don't have children/grandchildren	45/166	27	ref	0.657
no	16/52	31	1.14 (0.7 - 1.83)	
prefer not to say	10/50	20	0.74 (0.4 - 1.35)	
yes	15/55	27	1.01 (0.61 - 1.66)	
**Vaccinating children against COVID-19 may lower their risk of transmitting the virus to their family members**				
agree	14/54	26	ref	<0.001
disagree	2/36	5.6	0.21 (0.05 - 0.89)	
I don't know	5/47	11	0.41 (0.16 - 1.05)	
neither agree nor disagree	11/57	19	0.74 (0.37 - 1.49)	
strongly agree	43/77	56	2.15 (1.32 - 3.53)	
strongly disagree	11/52	21	0.82 (0.41 - 1.63)	
**Childhood COVID-19 vaccination offers protection against long-term health effects associated with the virus**				
agree	28/72	39	ref	<0.001
disagree	11/59	19	0.48 (0.26 - 0.88)	
I don't know	4/43	9.3	0.24 (0.09 - 0.64)	
neither agree nor disagree	6/49	12	0.31 (0.14 - 0.7)	
strongly agree	28/61	46	1.18 (0.79 - 1.76)	
strongly disagree	9/39	23	0.59 (0.31 - 1.13)	
**COVID-19 generally does not cause severe illness among children.**				
agree	12/48	25	ref	<0.001
disagree	12/54	22	0.89 (0.44 - 1.79)	
I don't know	8/46	17	0.7 (0.31 - 1.55)	
neither agree nor disagree	10/54	19	0.74 (0.35 - 1.56)	
strongly agree	8/49	16	0.65 (0.29 - 1.46)	
strongly disagree	36/72	50	2 (1.16 - 3.44)	

**Administering COVID-19 vaccines to children may help decrease the number of days they miss school**				
agree	40/88	46	ref	<0.001
disagree	10/43	23	0.51 (0.28 - 0.92)	
I don't know	9/48	19	0.41 (0.22 - 0.78)	
neither agree nor disagree	6/34	18	0.39 (0.18 - 0.83)	
strongly agree	17/59	29	0.63 (0.4 - 1.01)	
strongly disagree	4/51	7.8	0.17 (0.07 - 0.45)	
**COVID-19 may lead to severe illness or even death in children**				
agree	38/81	47	ref	<0.001
disagree	11/49	22	0.48 (0.27 - 0.85)	
I don't know	5/47	11	0.23 (0.1 - 0.54)	
neither agree nor disagree	10/44	23	0.48 (0.27 - 0.88)	
strongly agree	15/49	31	0.65 (0.4 - 1.06)	
strongly disagree	7/53	13	0.28 (0.14 - 0.58)	
**Vaccinating children is effective in reducing severity of COVID-19 if they infected**				
agree	27/69	39	ref	<0.001
disagree	6/41	15	0.37 (0.17 - 0.83)	
I don't know	6/46	13	0.33 (0.15 - 0.74)	
neither agree nor disagree	9/57	16	0.4 (0.21 - 0.79)	
strongly agree	33/67	49	1.26 (0.86 - 1.84)	
strongly disagree	5/43	12	0.3 (0.12 - 0.71)	
**Vaccinating children against COVID-19 is effective in reducing their chances of getting infected**				
agree	18/63	29	ref	<0.001
disagree	8/47	17	0.6 (0.28 - 1.25)	
I don't know	10/47	21	0.74 (0.38 - 1.46)	
neither agree nor disagree	4/49	8.2	0.29 (0.1 - 0.79)	
strongly agree	34/68	50	1.75 (1.11 - 2.76)	
strongly disagree	12/49	25	0.86 (0.46 - 1.61)	

Table S8: Practice factors influenced paediatricians recommending COVID-19 vaccine for children

Category	n/N	Prevalence (%)	Prevalence ratio (95% CI)	P value
**Are you involved in routine vaccination to children?**				
no, I am not directly involved, but I oversee vaccination programs in my practice	26/92	28	ref	0.6951
no, I am not involved in routine vaccinations for children	29/121	24	0.85 (0.54 - 1.34)	
yes, I am directly involved in administering routine vaccinations	31/110	28	1 (0.64 - 1.55)	
**How many children do you vaccinate monthly?**				
10-20 children	10/24	42	ref	0.021
21-50 children	10/28	36	0.86 (0.43 - 1.7)	
less than 10 children	10/32	31	0.75 (0.37 - 1.51)	
more than 50 children	1/26	3.8	0.09 (0.01 - 0.67)	
not applicable	55/213	26	0.62 (0.37 - 1.05)	
**What's your level of experience in administering routine vaccination to children?**				
experienced	6/20	30	ref	0.6779
limited experience	6/32	19	0.62 (0.23 - 1.67)	
no experience	58/210	28	0.92 (0.46 - 1.86)	
somewhat experienced	11/35	31	1.05 (0.46 - 2.4)	
very experienced	5/26	19	0.64 (0.23 - 1.8)	
**Are you involved in COVID-19 vaccination to children?**				
no, i am not involved in vaccine administration	45/163	28	ref	0.2822
yes, i administer vaccines directly	1/1	100	3.62 (2.82 - 4.64)	
yes, i oversee vaccine administration by other healthcare providers	40/159	25	0.91 (0.63 - 1.31)	
**How frequently do you recommend COVID-19 for children in your practice?**				
always	45/70	64	ref	<0.001
never	13/87	15	0.23 (0.14 - 0.4)	
occasionally	8/48	17	0.26 (0.13 - 0.5)	
often	12/48	25	0.39 (0.23 - 0.65)	
rarely	8/70	11	0.18 (0.09 - 0.35)	
**Have you perceived any changes in the level of parental concern in routine vaccinations?**				
no, it has not changed	16/66	24	ref	0.9612

yes, it has decreased a lot	17/59	29	1.19 (0.66 - 2.13)	
yes, it has decreased slightly	16/55	29	1.2 (0.66 - 2.17)	
yes, it has increased a lot	22/82	27	1.11 (0.63 - 1.93)	
yes, it has increased slightly	15/61	25	1.01 (0.55 - 1.87)	
**Do you trust the health authorities' recommendations on COVID-19 vaccination?**				
a little	15/114	13	ref	<0.001
a lot	41/70	59	4.45 (2.67 - 7.42)	
moderately	20/77	26	1.97 (1.08 - 3.61)	
not at all	10/62	16	1.23 (0.59 - 2.56)	
**Do you trust the WHO recommendations on COVID-19 vaccination?**				
a little	8/97	8.2	ref	<0.001
a lot	43/73	59	7.14 (3.58 - 14.25)	
moderately	20/76	26	3.19 (1.49 - 6.84)	
not at all	15/77	20	2.36 (1.06 - 5.28)	
**Has your trust in health authorities changed since COVID-19 pandemic?**				
decreased	29/106	27	ref	0.9755
increased	27/103	26	0.96 (0.61 - 1.5)	
remained unchanged	30/114	26	0.96 (0.62 - 1.49)	

Informed Consent sheet (English)

Information Sheet

Knowledge, Attitudes, and Practices of pediatricians towards COVID-19 vaccination in children

Principal Investigator: Dr. Yousef Mosleh

Introduction:

This information sheet is designed to provide you with information about a study among paediatricians in West Bank and what it involves. Please read the following carefully. If you have any questions or concerns, feel free to ask before making a decision to participate.

Purpose of the Study:

The Ministry of Health is conducting this study is to assess the knowledge, attitudes, and practices of paediatricians in the West Bank, Palestine, regarding COVID-19 vaccination in children. By participating, you will contribute valuable information that will help inform public health efforts aimed at ensuring the health and well-being of children in our community.

Study Procedures:

You will be contacted via telephone by a member of our research team. During the survey, you will be asked questions related to your knowledge, attitudes, and practices concerning COVID-19 vaccination in children. The survey is expected to take approximately 10-15 minutes of your time.

Confidentiality:
Your responses will be kept confidential and will not be linked to your identity. We will not collect any personal information that could identify you.
Data Security: All collected data will be securely stored and only authorized research personnel will have access to the data.

Participation in this study is entirely voluntary. You have the right to refuse to participate or to withdraw at any time without penalty. Your decision will not affect your medical care or professional standing in any way.

Consent:

By participating in this telephone survey, you indicate your informed consent to take part in the study.

If you agree to participate, please reply with "I consent" when our research team contacts you for the survey.

If you do not wish to participate, you may simply decline when contacted, and this will not have any negative consequences for you.

Thank you for considering participation in our study. Your contribution is greatly appreciated.

Contact Information:

If you have any questions or concerns about this study, you may contact the principal investigator at any time.
Dr. Yousef Mosleh
Email: Youssef.tarifi@gmail.com
Mobile/whatsapp: +972599151605

Informed consent sheet (Arabic)

موافقة على الاشتراك في بحث علمي

المعرفة والتوجهات والممارسات لأطباء الأطفال تجاه لقاح فيروس كوفيد-19 للأطفال

الباحث الرئيسي: الدكتور يوسف مصلح \ قسم الطب الوقائي\ وزارة الصحة الفلسطينية

مقدمة:

تم تصميم ورقة المعلومات هذه لتزويدك بمعلومات حول دراسة نجريها بين أطباء الأطفال في الضفة الغربية. يرجى قراءة ما يلي بعناية. إذا كان لديك أي أسئلة أو مخاوف، فلا تتردد في طرحها قبل اتخاذ قرار بالمشاركة.

غرض الدراسة:

تجري وزارة الصحة هذه الدراسة لتقييم المعرفة والتوجهات والممارسات لأطباء الأطفال في الضفة الغربية، فلسطين، تجاه لقاح فيروس كوفيد-19 للأطفال. من خلال المشاركة، ستقدم معلومات قيمة ستساهم في جهود الصحة العامة التي تهدف إلى ضمان صحة ورفاهية الأطفال في مجتمعنا.

إجراءات الدراسة:

سيتم الاتصال بك عبر الهاتف من قبل أحد أعضاء فريق البحث لدينا.
خلال الاستطلاع، سيُطلب منك الإجابة على أسئلة تتعلق بمعرفتك وتوجهاتك وممارساتك فيما يتعلق بتطعيم فيروس كوفيد-19 للأطفال. من المتوقع أن يستغرق الاستطلاع ما يقرب من 10-15 دقيقة من وقتك.

السرية:
سيتم الاحتفاظ بإجاباتك بسرية تامة ولن يتم ربطها بهويتك. لن نقوم بجمع أي معلومات شخصية يمكن أن تحدد هويتك.
أمان البيانات: ستتم مراعاة أمان جميع البيانات التي تم جمعها وسيتم الوصول إليها فقط من قبل الأفراد المخولين بالبحث.

المشاركة في هذه الدراسة تكون تمامًا اختيارية. لديك الحق في رفض المشاركة أو سحب مشاركتك في أي وقت دون عواقب سلبية. سيكون قرارك لا يؤثر على رعايتك الطبية أو وضعك المهني بأي شكل من الأشكال.

موافقة:

***Cont.* Informed consent sheet (Arabic)**

من خلال المشاركة في هذا الاستطلاع عبر الهاتف، تشير إلى موافقتك المستنيرة على المشاركة في الدراسة.

إذا وافقت على المشاركة، يُرجى الرد بـ "أنا موافق" عندما يتصل بك فريق البحث لدينا للإجراء الاستطلاع.

إذا كنت لا ترغب في المشاركة، يمكنك ببساطة رفض المشاركة عند الاتصال بك، ولن يكون لذلك أي تبعات سلبية بالنسبة لك.

نقدر لك مشاركتك في البحث بشكل كبير.

معلومات الاتصال:

إذا كان لديك أي أسئلة أو مخاوف حول هذه الدراسة، يمكنك الاتصال بالباحث الرئيسي في أي وقت.

الدكتور يوسف مصلح
البريد الإلكتروني: Youssef.tarifi@gmail.com
الهاتف المحمول / واتساب: 972599151605+

Questionnaire (En)

Knowledge, attitude and practice of paediatricians in West Bank, Palestine towards COVID-19 vaccination among children

Dear Participant,

We sincerely appreciate your willingness to participate in our study aimed at understanding the knowledge, attitudes, and practices of paediatricians in the West Bank, Palestine, regarding COVID-19 vaccination in children. Your insights and experiences are invaluable and will contribute to public health efforts aimed at ensuring the health and well-being of children in our community.

Your participation in this questionnaire is voluntary and confidential. Your responses will be anonymized, and no personal identifying information will be collected.

In this questionnaire, you will be asked a series of questions related to your knowledge, attitudes, and practices regarding COVID-19 vaccination among children. Additionally, we will collect some demographic and professional information to better understand your background.

Please answer each question to the best of your knowledge and experience. If you are uncertain about any question or prefer not to answer please feel free to indicate this to the interviewer.

Thank you for participation in our study. Your contribution is greatly appreciated.

Research team,

Sector 1: Demographic information

1.1 Gender
- □ Male
- □ Female

1.2 How old are you?

1.3 What is your marital status?
- □ Single
- □ Married
- □ Divorced
- □ Widowed

1.4 Do you have any children or grandchildren aged less than 15 years?
- □ Yes
- □ No

1.5 How many years have you worked as a paediatrician?

1.6 What is your primary place of practice?
- □ Private Sector
- □ Public sector
- □ Both Private and Public sectors
- □ Others (Please specify) ………………

1.7 At what type of healthcare facility do you mainly work?

- ☐ Hospital
- ☐ Clinic
- ☐ Private practice
- ☐ Others (Please specify) ___________________________

1.8 What is your primary specialty within paediatrics, if any?

- ☐ General paediatrics
- ☐ Paediatric Allergy and Immunology
- ☐ Paediatric Cardiology
- ☐ Paediatric Oncology
- ☐ Paediatric Infectious Diseases
- ☐ Paediatric Neurology
- ☐ Paediatric Surgery
- ☐ Paediatric Endocrinology
- ☐ Paediatric Pulmonology
- ☐ Other (please specify) ___________________________________

1.9 Do you regularly attend continuing medical education (CME) or professional development programs?

- ☐ Yes
- ☐ No

1.10 Have you ever been infected with COVID-19?

- ☐ Yes
- ☐ No

1.11 Have you received the COVID-19 vaccine?

- ☐ Yes, I'm fully vaccinated.
- ☐ Yes, but I have only received one dose.
- ☐ No, I have not received the vaccine.

1.12 Would you be willing to receive a COVID-19 vaccine on a regular basis if recommended by health authorities?

- ☐ Yes, I would be willing to receive it regularly.
- ☐ No, I would not be willing to receive it regularly.
- ☐ Unsure/Depends on the recommendations.

1.13 Do you have any underlying medical conditions or health concerns that may affect your work or health status? (e.g., chronic illnesses, allergies, etc.)

- ☐ Yes
- ☐ No
- ☐ Prefer not to say

1.14 Do you have any children or grandchildren below 18 years of age?

- ☐ Yes
- ☐ No. *If NO, go to question 1.17*
- ☐ 1.15 If YES, have any of your own children or grandchildren below 18 years of age ever been infected with COVID-19?
- ☐ Yes
- ☐ No
- ☐ Not applicable. I do not have children or grandchildren.

1.16 If YES to question 1.14, have any of your own children or grandchildren below 18 years of age received a COVID-19 vaccine?

- ☐ Yes
- ☐ No

1.17 Have you received any formal training or updates regarding COVID-19 vaccination in children?

- ☐ Yes
- ☐ No

Section 2: Knowledge about COVID-19 vaccination in children

2.1 Does WHO recommend vaccination against COVID-19 in children 5 years old and above?

- ☐ Yes
- ☐ No
- ☐ I don't know

2.2 If yes, which COVID-19 vaccine(s) does the WHO recommend for children? (Open-ended)

2.3 What age range does the Palestinian Ministry of Health recommend for COVID-19 vaccination in children, according to your knowledge?

- ☐ 5 years and above
- ☐ 12 years and above
- ☐ I am not aware of the Palestinian Ministry of Health's recommendations
- ☐ Other (please specify)_________________________________

2.4 Which COVID-19 vaccine(s) have been authorized for use in children in the West Bank, Palestine?

2.5 Please rate your knowledge about the safety and efficacy of COVID-19 vaccines for children on a scale of 1 to 5, with 1 being very low knowledge and 5 being very high knowledge.

- ☐ 1 (Very Low)
- ☐ 2 (Low)
- ☐ 3 (Moderate)
- ☐ 4 (High)
- ☐ 5 (Very High)

2.6 What are some common side effects associated with COVID-19 vaccination in children? (Open-ended)

2.7 Children previously infected with SARS-CoV-2 can safely receive COVID-19 vaccine

- ☐ Yes
- ☐ No
- ☐ I don't know

2.8 COVID-19 vaccine is safe to administer with other childhood vaccines (MMR, OPV, IPV, BCG, DPT)

- ☐ Yes
- ☐ No
- ☐ I don't know

Section three: Attitudes towards COVID-19 in children

3.1 Do you agree with mandating vaccination of children against COVID-19?

- ☐ No
- ☐ Yes, but only for children at risk of severe illness
- ☐ Yes, for all children over a certain age (please specify the age): .....

- ☐ Yes, for all children

3.2 How confident are you in the safety of COVID-19 vaccines for children?

- ☐ Very confident
- ☐ Somewhat confident
- ☐ Neutral
- ☐ Somewhat hesitant
- ☐ Very hesitant

3.3 What factors, if any, make you hesitant about recommending COVID-19 vaccines for children? (Select all that apply)

- ☐ Safety concerns
- ☐ Efficacy concerns
- ☐ Lack of data on long-term effects
- ☐ Parental vaccine hesitancy
- ☐ Other (please specify)

3.4 If you have not already given COVID-19 vaccination to your own children, would you be willing to do so in the future (if applicable)?

- ☐ Yes, I would be willing to give COVID-19 vaccination to my children.
- ☐ No, I would not be willing to give COVID-19 vaccination to my children.
- ☐ I do not have children.
- ☐ Prefer not to say

3.5 Please indicate your level of agreement or disagreement with the following statements about COVID-19 and COVID-19 vaccination. If you do not know, please let me know.

Statement	Strongly Disagree	Disagree	Neither Agree nor Disagree	Agree	Strongly Agree	I Don't Know
COVID-19 generally does not cause severe illness among children.						
COVID-19 may lead to severe illness or even death in children.						
Vaccinating children is effective in reducing their chances of getting COVID-19.						
Vaccinating children is effective in reducing the severity of COVID-19 if they infected with the virus						
Administering COVID-19 vaccines to children may help decrease the number of days they miss school.						
Vaccinating children against COVID-19 may lower their risk of transmitting the virus to their family members.						
Childhood COVID-19 vaccination offers protection against long-term health effects often associated with the virus (long COVID).						

Section 4: Practices and trust related to COVID-19 vaccination in children

4.1 Are you actively involved in administering routine vaccinations to children in your practice?

- ☐ Yes, I am directly involved in administering routine vaccinations.
- ☐ No, I am not directly involved, but I oversee vaccination programs in my practice.
- ☐ No, I am not involved in routine vaccinations for children.

4.2 If yes, approximately how many children do you vaccinate monthly as part of your routine vaccination services?

- ☐ Less than 10 children
- ☐ 10-20 children
- ☐ 21-50 children
- ☐ More than 50 children

4.3 Please indicate your level of experience in administering routine vaccinations to children.

- ☐ Very Experienced
- ☐ Experienced
- ☐ Somewhat Experienced
- ☐ Limited Experience
- ☐ No Experience

4.4 Are you involved in the administration of COVID-19 vaccines to children in your healthcare facility?

- ☐ Yes, I administer vaccines directly.
- ☐ Yes, I oversee vaccine administration by other healthcare providers.
- ☐ No, I am not involved in vaccine administration.

4.5 How frequently do you recommend COVID-19 vaccination for eligible children in your practice?

- ☐ Always
- ☐ Often
- ☐ Occasionally
- ☐ Rarely
- ☐ Never

4.6 Since the start of the COVID-19 pandemic, have you perceived any changes in the level of parental concern about vaccine safety and/or effectiveness:

- ☐ Yes, it has increased a lot
- ☐ Yes, it has increased slightly
- ☐ No, it has not changed
- ☐ Yes, it has decreased slightly
- ☐ Yes, it has decreased a lot

4.7 Do you trust the health authorities' recommendations on COVID-19 vaccination?

- ☐ Not at all
- ☐ A little
- ☐ Moderately
- ☐ A lot

4.8 Do you trust the WHO recommendations on COVID-19 vaccination?

- ☐ Not at all
- ☐ A little
- ☐ Moderately
- ☐ A lot

4.9 After the COVID-19 pandemic, has your trust in health authorities:

- ☐ Decreased
- ☐ Remained unchanged
- ☐ Increased

Do you have any additional comments or insights related to COVID-19 vaccination in children that you would like to share? (Open-ended)

Thank you for completing this survey. If you have any further questions or concerns, please feel free to contact us.

[End of Questionnaire]

Questionnaire (Ar)

المعرفة والاتجاه والممارسة لدى أطباء الأطفال في الضفة الغربية، فلسطين تجاه تلقيح كوفيد-19 للأطفال

عزيز المشارك،

نحن نقدر بصدق استعدادك للمشاركة في دراستنا التي تهدف إلى فهم المعرفة والاتجاهات والممارسات لدى أطباء الأطفال في الضفة الغربية، فلسطين، بشأن تلقيح كوفيد-19 للأطفال. تجاربك وآراؤك لها قيمة كبيرة وستسهم في الجهود الصحية العامة التي تهدف إلى ضمان صحة ورفاهية الأطفال في مجتمعنا.

مشاركتك في هذا الاستبيان هي طوعية وسرية. سيتم تجميع إجاباتك بشكل مجهول، ولن يتم جمع أي معلومات تحدد هويتك الشخصية.

في هذا الاستبيان، ستُطلب منك سلسلة من الأسئلة المتعلقة بمعرفتك واتجاهاتك وممارساتك بشأن تلقيح كوفيد-19 للأطفال. بالإضافة إلى ذلك، سنجمع بعض المعلومات الديموغرافية والمهنية لفهم خلفيتك بشكل أفضل.

يرجى الرد على كل سؤال بأفضل معرفتك وتجربتك. إذا كنت غير متأكد من أي سؤال أو تفضلت بعدم الرد، فلا تتردد في الإشارة إلى ذلك للمقابل.

شكرًا لمشاركتك في دراستنا. تقديرنا لمساهمتك كبير.

فريق البحث،

القسم 1: المعلومات الديموغرافية

1.1الجنس

- ○ ذكر
- ○ أنثى

1.2كم عمرك؟

1.3ما هو وضعك الزوجي؟

- أعزب
- متزوج
- مطلق
- أرمل

1.4هل لديك أطفال أو أحفاد تقل أعمارهم عن 15 عامًا؟

- نعم
- لا

1.5كم عدد سنوات عملك كطبيب أطفال؟

1.6ما هو مكان ممارستك الرئيسي؟

- القطاع الخاص
- القطاع العام
- القطاعين الخاص والعام
- آخر (ارجو التحديد)

1.7في أي نوعٍ من مرافق الرعاية الصحية تعمل أساسًا؟

- مستشفى
- عيادة
- ممارسة خاصة
- آخر (ارجو التحديد)

1.8ما هو التخصص الرئيسي لديك في طب الأطفال، إن كان هناك؟

- طب الأطفال العام
- طب الأطفال والحساسية والمناعة
- طب الأطفال وأمراض القلب
- طب الأطفال وأمراض الأورام
- طب الأطفال وأمراض العدوى
- طب الأطفال والأعصاب
- جراحة الأطفال
- طب الأطفال وأمراض الغدة

طب الأطفال وأمراض الجهاز التنفسي

- آخر (ارجو التحديد)

1.9هل تحضر بانتظام برامج التعليم الطبي المستمر (CME) أو برامج التطوير المهني؟

- o نعم
- o لا

1.10هل أصيبت بكوفيد-19 في وقت سابق؟

- o نعم
- o لا

1.11هل تلقيت لقاح كوفيد-19؟

- o نعم، تلقيت اللقاح بالكامل.
- o نعم، لكن لم أتلق سوى جرعة واحدة.
- o لا، لم أتلق اللقاح.

1.12هل ستكون على استعداد لتلقي لقاح كوفيد-19 بانتظام إذا تم توصية به من قبل السلطات الصحية؟

- o نعم، سأكون على استعداد لتلقيه بانتظام.
- o لا، لن أكون على استعداد لتلقيه بانتظام.
- o غير متأكد/يعتمد على التوصيات.

1.13هل لديك أية حالات طبية أساسية أو مخاوف صحية قد تؤثر على عملك أو حالتك الصحية؟ (مثل الأمراض المزمنة، الحساسية، إلخ(.

- o نعم
- o لا
- o أفضل عدم الإفصاح
- o

1.14هل لديك أطفال أو أحفاد تقل أعمارهم عن 18 عامًا؟

- o نعم
- o لا. إذا كانت الإجابة "لا"، انتقل إلى السؤال 1.17

1.15إذا كانت الإجابة "نعم" في السؤال 1.14، هل أصيب أحد أطفالك أو أحفادك الخاصين بعمر دون 18 عامًا بكوفيد-19؟

- o نعم
- o لا

1.16إذا كانت الإجابة "نعم" في السؤال 1.14، هل تلقى أحد أطفالك أو أحفادك الخاصين دون سن 18 عامًا لقاح كوفيد-19؟

- نعم
- لا

1.17هل تلقيت أي تدريب رسمي أو تحديثات بشأن تلقيح كوفيد-19 للأطفال؟
- نعم
- لا

القسم 2: المعرفة حول تلقيح كوفيد-19 للأطفال

2.1هل توصي منظمة الصحة العالمية بتلقيح الأطفال الذين تبلغ أعمارهم 5 سنوات وما فوق ذلك؟
- نعم
- لا
- لا أعرف

2.2إذا كانت الإجابة "نعم"، أي لقاحات كوفيد-19 يوصي منظمة الصحة العالمية بها للأطفال؟ (اجابة مفتوحة(

2.3وفقًا لمعرفتك، ما هو النطاق العمري الذي توصي به وزارة الصحة الفلسطينية لتلقيح الأطفال ضد كوفيد-19؟
- 5سنوات وما فوق
- 12سنة وما فوق
- لا أعرف توصيات وزارة الصحة الفلسطينية
- آخرون (الرجاء تحديد__________________________ (

2.4ما هي اللقاحات المصرح بها لاستخدامها في الأطفال في الضفة الغربية، فلسطين؟

2.5يُرجى تقييم معرفتك بشأن سلامة وفعالية لقاحات كوفيد-19 للأطفال على مقياس من 1 إلى 5، حيث يكون 1 معرفة منخفضة جدًا و5 معرفة عالية جدًا.

- 1(معرفة منخفضة جدًا)

- 2(معرفة منخفضة)
- 3(معرفة متوسطة)
- 4(معرفة عالية)
- 5(معرفة عالية جدًا)

2.6ما هي بعض الآثار الجانبية الشائعة المرتبطة بتلقيح كوفيد-19 للأطفال؟ (اجابة مفتوحة(

2.7هل يمكن للأطفال الذين أصيبوا سابقًا بفيروس SARS-CoV-2 تلقي لقاح كوفيد-19 بأمان؟

- نعم
- لا
- لا أعرف

2.8هل يمكن إعطاء لقاح كوفيد-19 بأمان مع لقاحات الطفولة الأخرى (جدري الماء والشلل النقال وشلل الأطفال والسل والسعال الديكي والكزاز.. الخ)؟

- نعم
- لا
- لا أعرف

القسم الثالث: الاتجاهات تجاه كوفيد-19 لدى الأطفال

3.1هل توافق على إلزام تلقيح الأطفال ضد كوفيد-19؟

- لا
- نعم، ولكن فقط للأطفال الذين يعانون من خطر مرتفع للمرض الشديد
- نعم، لجميع الأطفال فوق سن معين (الرجاء تحديد العمر..... :(
- نعم، لجميع الأطفال

3.2كم هو ثقتك في سلامة لقاحات فيروس كوفيد-19 للأطفال؟

- ثقة كبيرة
- ثقة إلى حد ما
- محايد
- تردد إلى حد ما
- تردد كبير

3.3ما هي العوامل التي تجعلك تتردد في توصية بلقاحات فيروس كوفيد-19 للأطفال؟ (اختر كل ما ينطبق(

- o مخاوف من السلامة
- o مخاوف من الفعالية
- o نقص البيانات حول التأثيرات على المدى الطويل
- o تردد الآباء في تلقي اللقاح
- o غير ذلك (الرجاء التوضيح(

3.4إذا لم تقم بالفعل بإعطاء لقاح فيروس كوفيد-19 لأطفالك الخاصين، هل ستكون مستعدًا للقيام بذلك مستقبلاً (إذا كان ذلك ممكنًا)؟

- o نعم، سأكون مستعدًا لإعطاء لقاح فيروس كوفيد-19 لأطفالي.
- o لا، لن أكون مستعدًا لإعطاء لقاح فيروس كوفيد-19 لأطفالي.
- o ليس لدي أطفال.
- o تفضل عدم الرد

3.5الرجاء الإشارة إلى مدى اتفاقك أو عدم اتفاقك مع البيانات التالية حول فيروس كوفيد-19 ولقاحاته. إذا كنت لا تعرف الإجابة، الرجاء إخباري.

لا أعرف	أوافق جداً	أوافق	لا أوافق ولا أعارض	أخالف	بيان أخالف جداً	
						فيروس كوفيد-19 عموما لا يسبب مرضًا خطيرًا بين الأطفال.
						فيروس كوفيد-19 يمكن أن يؤدي إلى مرض خطير أو حتى الوفاة بين الأطفال.
						تطعيم الأطفال فعّال في تقليل فرص إصابتهم بفيروس كوفيد-19.
						تطعيم الأطفال فعّال في تقليل شدة فيروس كوفيد-19 إذا أصيبوا بالفيروس.
						إعطاء لقاحات فيروس كوفيد-19 للأطفال قد

يساعد في تقليل عدد الأيام التي يتغيبون فيها عن المدرسة.						
تطعيم الأطفال ضد فيروس كوفيد-19 قد يقلل من خطر نقل الفيروس لأفراد الأسرة.						
تطعيم الأطفال ضد فيروس كوفيد-19 يقدم حماية ضد التأثيرات الصحية طويلة الأمد التي ترتبط غالبًا بالفيروس (كوفيد طويل الأمد.(						

.

القسم الرابع: الممارسات والثقة المتعلقة بتطعيم فيروس كوفيد-19 للأطفال

4.1هل أنت مشارك في إعطاء اللقاحات الروتينية للأطفال في ممارستك الطبية؟

- o نعم، أنا مشارك مباشرة في إعطاء اللقاحات الروتينية.
- o لا، أنا لست مشاركًا مباشرة، ولكني أراقب برامج التطعيم في ممارستي.
- o لا، أنا لست مشاركًا في التطعيم الروتيني للأطفال.

4.2إذا كان الجواب نعم، فكم عدد الأطفال الذين تطعّمهم شهريًا ضمن خدمات التطعيم الروتينية الخاصة بك تقريبًا؟

- o أقل من 10 أطفال
- o 10-20طفلاً
- o 21-50طفلاً
- o أكثر من 50 طفلاً

4.3الرجاء الإشارة إلى مدى خبرتك في إعطاء اللقاحات الروتينية للأطفال.

- خبرة كبيرة جدًا
- خبرة
- خبرة إلى حد ما
- خبرة محدودة
- لا توجد خبرة

4.4هل أنت مشارك في إعطاء لقاحات فيروس كوفيد-19 للأطفال في مرفق الرعاية الصحية الخاص بك؟

- نعم، أعطي اللقاحات مباشرة.
- نعم، أنا أراقب إعطاء اللقاحات من قبل مقدمي الرعاية الصحية الآخرين.
- لا، لست مشاركًا في إعطاء اللقاح.

4.5مدى توصيتك بتطعيم الأطفال المؤهلين بلقاح فيروس كوفيد-19 في ممارستك؟

- دائمًا
- غالبًا
- أحيانًا
- نادرًا
- أبدًا

4.6منذ بداية جائحة كوفيد-19، هل لاحظت أي تغيير في مستوى قلق الآباء بشأن سلامة اللقاحات الروتينية للأطفال و/أو فعاليتها:

- نعم، زاد كثيراً
- نعم، زاد قليلاً
- لا، لم يتغير
- نعم، انخفض قليلاً
- نعم، انخفض كثيراً

4.7هل تثق في توصيات السلطات الصحية بشأن تطعيم فيروس كوفيد-19؟

- على الإطلاق لا
- قليلاً
- بشكل معتدل
- كثيرًا

4.8هل تثق في توصيات منظمة الصحة العالمية بشأن تطعيم فيروس كوفيد-19؟

- على الإطلاق لا

- قليلاً
- بشكل معتدل
- كثيرًا

4.9بعد جائحة كوفيد-19، هل انخفضت ثقتك في سلطات الصحة:

- انخفضت
- لم تتغير
- زادت

هل لديك تعليقات أو رؤى إضافية تتعلق بتطعيم فيروس كوفيد-19 للأطفال ترغب في مشاركتها؟ (إجابة مفتوحة)

شكرًا لاستكمال هذا الاستبيان. إذا كان لديك أي أسئلة أو مخاوف إضافية، فلا تتردد في الاتصال بنا.

[نهاية الاستبيان]
